# Using liner surface modes in acoustic ducts to make obstacles reflectionless

**DOI:** 10.1038/s41598-019-43538-3

**Published:** 2019-05-06

**Authors:** Maaz Farooqui, Yves Aurégan, Vincent Pagneux

**Affiliations:** Laboratoire d’Acoustique de l’Université du Mans, Centre National de la Recherche Scientifique (CNRS), Le Mans Université, Avenue Olivier Messiaen, 72085 Le Mans, Cedex, 9 France

**Keywords:** Acoustics, Microwave photonics

## Abstract

Acoustic cloaking for the suppression of backscattering inside ducts is proposed in the audible range where plane waves are curved around the object using the surface modes of the liner. It is numerically shown that a slowly varying resonant liner (e.g. based on an array of tubes) creates a zone of silence in which an object of arbitrary shape can be acoustically hidden for a wide range of frequencies. And then, a resonant liner has deflecting properties without reflection of the wavefront, which are close to an ideal invisibility cloak. This kind of cloaking is effective in a wide frequency band and the cloaking band is a function of the impedance and height of the obstacle relative to the conduit. For smooth shaped obstacles, there is an ability of the object to help hide itself, which increases the cloaking frequency band (self-cloaking). Dispersion effects lead to slow sounds and distortion of the wave phase.

## Introduction

Hiding an obstacle from the scattering of electromagnetic, acoustic or elastic waves has recently been a subject of particular interest because of its promising applications^1–5^. These cloaking devices can be used not only for wave stealth, but also to protect the obstacle from wave effects^6^. However, the manufacturing of these cloaks and the widening of the effective frequency band still pose difficulties. These problems can be greatly reduced in some specific situations^7^. For instance, when the obstacle is located near a reflective wall, a carpet cloaking can be placed over this obstacle so that the waves are reflected as if there were no obstacle in front of the wall^8^. In this case, the metamaterials used to make the carpet no longer need to have highly anisotropic effective parameters with quasi-singular values, as in the general case, which facilitates their practical realization^9^. Another case where geometry can help cloaking is when an obstacle is located inside a waveguide. The general idea is to transform, gradually and without any scattering, the propagative wave into a surface wave at the wall of the waveguide^10,11^, which allows the wave to avoid the obstacle. This idea was first shown in the case of microwaves^10^ and it has also be widely applied in spoof plasmon waveguide^12–15^. In this paper, we propose to use for the first time a similar concept in acoustics.

**Figure 1:**
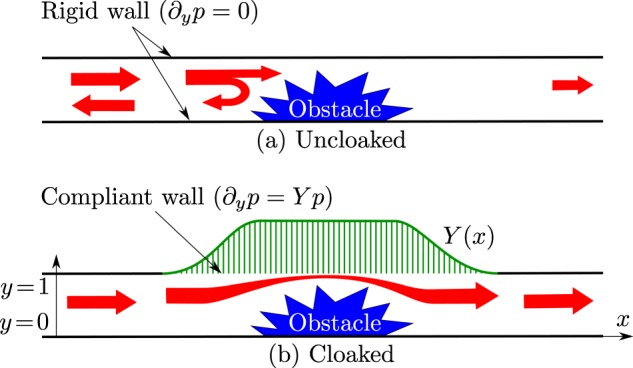
Schematic description of an obstacle in a duct with admittance (**a**) Uncloaked means a hard duct containing an arbitrary obstacle, (**b**) Cloaked means the same duct supplemented by a liner (compliant wall).

In acoustics,“liners” refer to acoustic materials located on the walls of the ducts. They act according to two distinct principles: the absorption of acoustic energy by visco-thermal effects and the scattering of acoustic waves by variations in the properties or geometry of the material that can occur even with non-dissipative liners^16^. Therefore, playing with this reactive part of the liner can lead to unusual properties, such as the acoustic cloaking of an obstacle in a duct. To achieve this, we consider a wall made with a large number of small lossless tubes, see Fig. 1. When the wavelength is large with respect to the transverse size of the tubes, the acoustic response of the wall can be described by the homogenized parameter that is the impedance or its inverse: the admittance.

In this paper, we first show that, by using a smooth axial variation of the wall impedance, it is possible to create a zone of silence in a duct. The purely reactive impedance gradually transforms a plane acoustic wave in the duct into an acoustic surface wave. This results in a bending of the acoustic wave towards the liner with a slower effective propagation velocity. Any obstacle placed in this silence zone will have no effect on wave propagation and becomes acoustically undetectable (as well as protected from wave effects) over a wide range of frequencies. A numerical study shows the cloaking of obstacles of different shapes. In addition, we show that an obstacle with a smooth cross section variation can be cloaked even if it is outside the silence zone. This effect is called self-cloaking. The last section of the paper is devoted to the limitations of the presented model. First, we find the necessary conditions to describe a discrete set of tubes by a homogenized impedance and then we show that the cloaking effect can be preserved even when there are losses in the tubes.

## Cloaking in Ducts Using the Wall Admittance

We consider the sound propagation in a two dimensional channel, see Fig. 1. The lower wall is rigid while the upper wall is compliant and described by a varying admittance *Y*(*x*). When the distances are non-dimensioned by the height of the channel *H*, the Helmholtz equation, governing the propagation of the acoustic pressure *p*, is:

1 $${\rm{\Delta }}p+{k}^{2}p=0$$

where *k* = *ωH*/*c*_0_ is the Helmholtz number, *ω* is the frequency and *c*_0_ is the sound velocity. The boundary conditions are ∂_*y*_*p* = 0 for *y* = 0 and ∂_*y*_*p* = *Yp* for *y* = 1. For an uniform admittance *Y*, the solution is searched under the form *p* = *A*cosh(*αy*)exp(i(−*ωt* + *βx*)) where *α*^2^ = *β*^2^ − *k*^2^ and this leads to the dispersion relation

2 $$Y=\alpha \,\tanh (\alpha )$$

**Figure 2:**
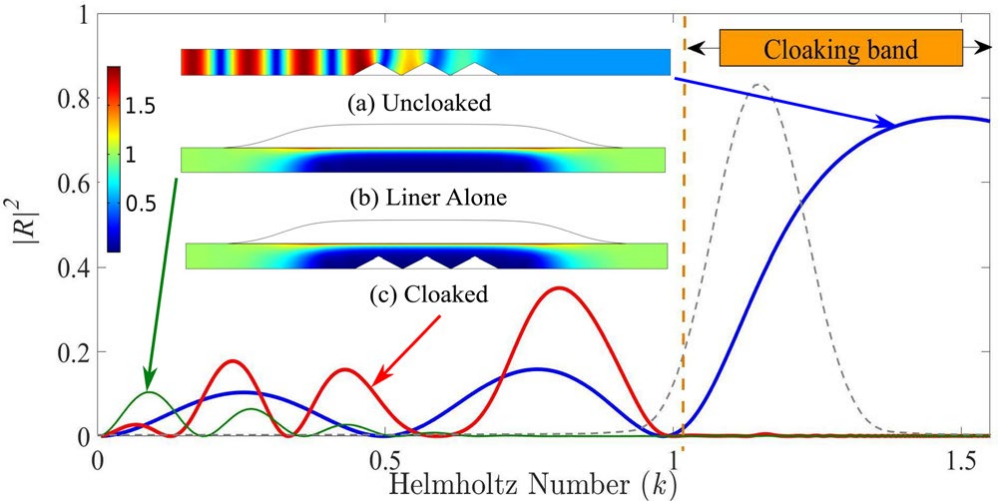
Reflection coefficient in energy |*R*|^2^ for an array of triangular obstacles in the uncloaked (blue), Lined duct (green) and cloaked (red) cases. (**a**) Absolute pressure field for the uncloaked case at *k* = 1.38, (**b**) Absolute pressure for lined duct at *k* = 1.38 and (**c**) Absolute pressure for the cloaked case at *k* = 1.38. The grey dotted line is the Fourier transform in arbitrary unit of the pulse used in Time domain section.

For a rigid wall, *Y* = 0, at low frequencies, only the plane wave can propagate thus *α* = 0 when *k* < *π*. If the admittance is positive and, is slowly varying compared to the sound wavelength, the local value of *α* increases progressively as *Y* increases. It means that the wave is more and more concentrated against the wall, see Fig. 2(b). All the computations have been made using Finite element based software “COMSOL” where a plane wave of unity amplitude (forced boundary condition) is incident from the left side of the duct and non-reflective conditions are used on both side of the duct A silence zone is then created near the wall opposite to the admittance. Any obstacle located in this silence zone has no influence on wave propagation. This obstacle is invisible in the sense that it does not produce any wave reflection. In order to verify the cloaking efficiency with a sharp scatterer, Fig. 2 depicts the reflected energy |*R*|^2^ for a series of triangular obstacles. The cloaking band in this case is from 1 ≤ *k* ≤ *π*/2. On this example, the reflection coefficient of the uncloaked obstacle (Fig. 2(a)) is |*R*| = 0.85 for *k* = 1.38 while when the admittance is present without any obstacle (Fig. 2(b)) or even with a scattering obstacle (Fig. 2(c)) it is reduced to |*R*| = 0.02 in the cloaking band. In the sequel, the cloaking zone is defined as the frequency band where |*R*| ≤ 0.1.

### Frequency dependence

The liner can realized by using small closed tubes perpendicular to the upper wall with variable lengths *b*(*x*). Neglecting viscothermal losses, the admittance is written

3 $$Y(x,k)=k\,\tan (k\,b(x)).$$

When *kb* ≪ 1, the admittance can be approximated by *Y* = *k*^2^*b* and the phase velocity of the wave is given by *c*_*φ*_ = (1 + *b*)^−1/2^ meaning that the wave velocity is reduced compared to sound velocity^16^. When the frequency *k* goes to the first resonant frequency of the tubes given by *k*_*r*_*b* = *π*/2, the admittance and *α* go to ∞. Thus, for frequencies slightly below *k*_*r*_, the wave decreases exponentially from the wall and has been transformed into a surface wave. Near *k*_*r*_, the phase velocity and the effective wavelength of the wave goes linearly to 0. The propagation is then highly dispersive.

The admittance profile is taken under the form

4 $$b(x)=\frac{{b}_{0}}{2}\,(\tanh (\frac{x+l}{d})-\,\tanh (\frac{x-l}{d}))$$

where the maximum tube height has been arbitrarily fixed to *b*_0_ = 1 and the two parameters *l* and *d* allow to settle separately the length of the admittance zone and maximal slope of the axial change in admittance. Those parameters are fixed in the following to *l* = 6 and *d* = 2 which correspond to an admittance variation smooth enough to avoid reflection for frequencies larger than *k* = *π*/4.

**Figure 3:**
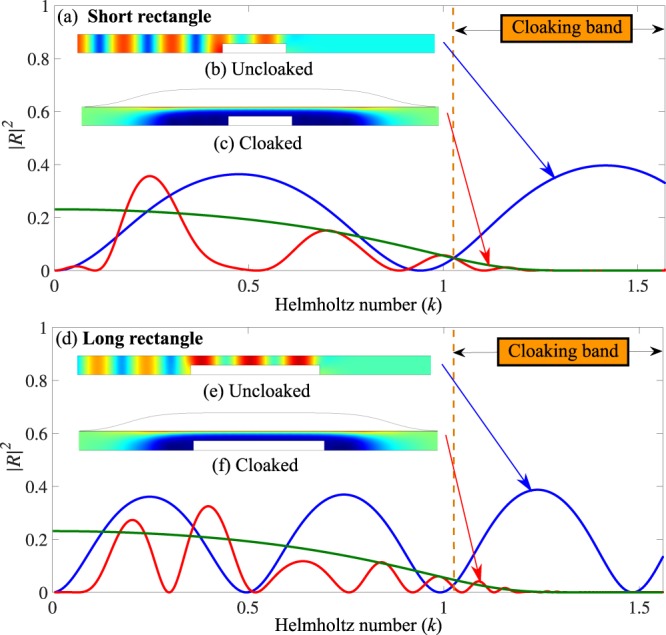
Comparison of the cloaking for short and long rectangular obstacles: (**a**–**c**) Short obstacle (**d**–**f**) Long obstacle. (**a**) and (**d**) |*R*|^2^ for uncloaked (blue) and cloaked (red) cases (*l* = 6, *d* = 2, *b*_0_ = 1). (**b**,**c**,**e** and **f**) Absolute pressure field for *k* = 1.38. The green curves in (**a**) and (**d**) is the value |2*R*_∞_|^2^ that forms the envelope for case with uniform admittance treatment on an infinite rectangular obstacle.

Two rectangular obstacles with the same height (*h*_0_ = 0.5) but different lengths (*w* = 3 and 6) are put in the shadow zone, see Fig. 3. For the short obstacle, the reflection coefficient at *k* = 1.38 is reduced from |*R*| = 0.63 to |*R*| = 0.0018 with the admittance. The reflection coefficient is reduced by the same amount for the long obstacle.

Without admittance, the oscillations of the reflection coefficient came from the interference of the waves reflected by the ascending step and by the descending step. These two waves have the same amplitude and then |*R*| is maximized by two times the reflection on the ascending step. On the same way, with admittance, |*R*| is maximized by 2|*R*_∞_|, plotted in green on Fig. 3(a,d), where *R*_∞_ is the reflection coefficient of an ascending step in presence of a uniform admittance on the upper wall that can be easily computed by a multimodal method. In the vicinity of the cloaking band, 2|*R*_∞_| is a good envelope of the oscillations of |*R*| with admittance and can be used to evaluate with simple calculation the lower frequency limit of the cloaking band. The upper frequency of the cloaking band is given by the tube resonance (*kb* = *π*/2).

This example shows that any object located in the shadow zone produces a small reflection on a broad frequency band even if this object covers the substantial surface of the shadow zone. An envelope of |*R*| near and in the cloaking band can be easily computed from the admittance and from the maximum height of the object.

## Time Domain

To illustrate the broadband capability of this cloaking effect, a time simulation of the configuration shown in the frequency domain on Fig. 2 is performed using COMSOL. The obstacle of total length *w* = 6 is made of 3 identical triangles of height *h*_0_ = 0.5. This particular shape of the obstacle is chosen to be an efficient Bragg scatterer for frequencies such as *kd* = *nπ*, where *n* is an integer and *d* = 2 is the distance between two triangles. As a result, in the uncloaked case depicted in Fig. 2, the reflection is large in the cloaking band (slightly below *k* = *π*/2). The admittance shape is given by Eq. 4 with *b*_0_ = 1, *l* = 6 and *d* = 2.

**Figure 4:**
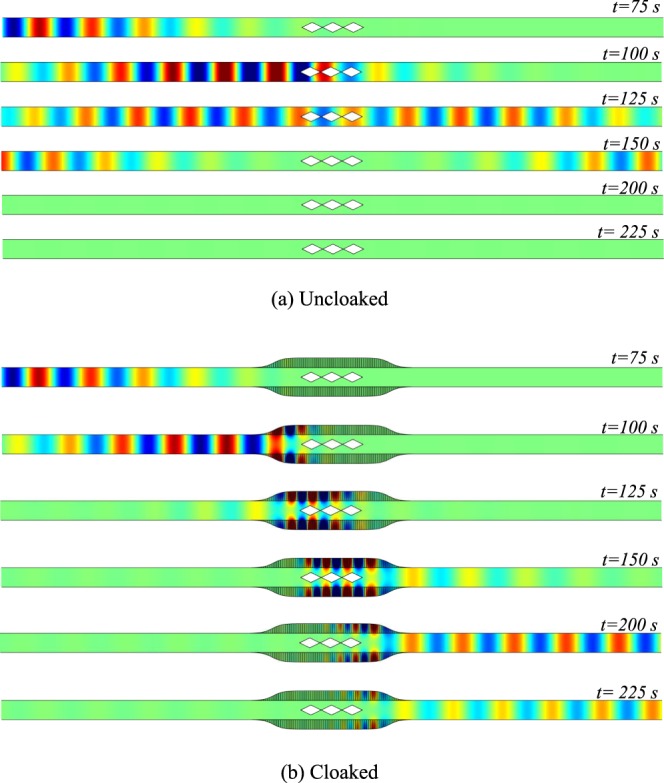
Time-domain results for pressure field with Gaussian-shaped wave packet on the left side of duct (**a**) Uncloaked, (**b**) Cloaked.

A Gaussian wave packet exp(−((*t* − *t*_0_)/*s*_*t*_)^2^/2)sin(*k*_*c*_*t*) is incident from the left side of the duct. The Fourier transform of this wave packet with a central frequency of *k*_*c*_ = 1.15 and with *s*_*t*_ = 13 is plotted in grey dotted line on Fig. 2 and is within the cloaked band. The pressure field at different time steps is shown for uncloaked and cloaked duct in Fig. 4(a,b). It can be noted that for better visualization, the obstacle and wall have been doubled and that the field has also been computed inside the tubes.

**Figure 5:**
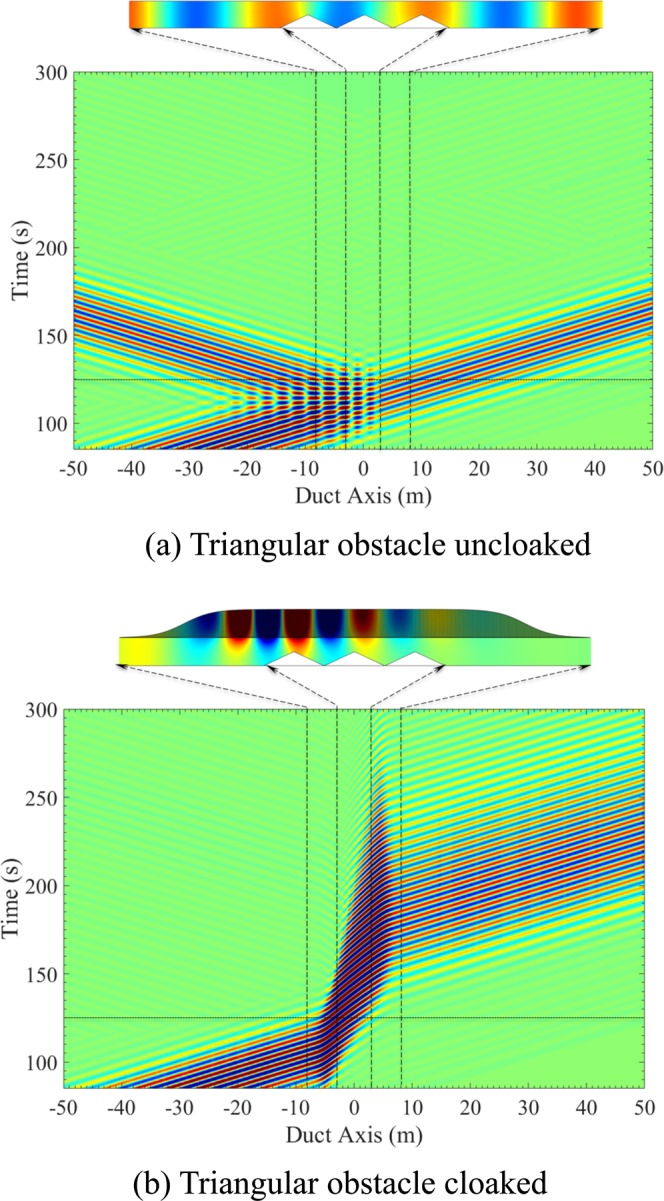
Time-domain results for the pressure averaged over the channel with Gaussian-shaped wave packet on the left side for a duct with triangular obstacle (**a**) Uncloaked, (**b**) Cloaked. The dotted horizontal line represents the time for which the pressure field is plotted.

A spatio-temporal representation of the pressure averaged over the channel is plotted in Fig. 5(a,b), for uncloaked and cloaked cases. The wave reflection is clearly visible in the uncloaked case and has disappeared in the cloaked case. In the cloaked case, the slowing down of sound when it enters the cloaked region is visible. The slope of the contour lines shows that, in the cloaking region, the sound propagation velocity is reduced by a factor of 2.5 on an average, while it propagates with a normalized sound velocity of *c*_0_ = 1 elsewhere. And so, by passing through the cloaking region, the sound wave is subject to a time delay compared to the uncloaked case. The wave dispersion, resulting from the change in the effective velocity with frequency, is illustrated in Fig. 5(b) by the widening of the transmitted wave packet. This effect is particularly significant in the present case because, in order to have a small time window for a better visualization, we have widened the frequency band in a region where the variation of the wave velocity as a function of frequency is very important.

## Beyond the Shadow Zone: Self-Cloaking

As mentioned in previous sections, the deviation of the wave towards the compliant wall is controlled by the parameter *α* which gives the exponential decay of the pressure from the wall. This parameter is directly related to the ratio *b* between the tubes height and the channel height. The height of the channel *H*_0_ between the obstacle and the liner, decreases with increasing height of the obstacle. A local dispersion relation (Eqn. 2) can be written in the form *k*tan(*kb*) = *α*tanh(*αH*_0_). The decrease in *H*_0_, increases the local *α* and therefore the presence of the obstacle helps to concentrate the wave towards the compliant wall. This effect is called self-cloaking and it improves the efficiency of the cloaking for smoothly varying obstacle.

**Figure 6:**
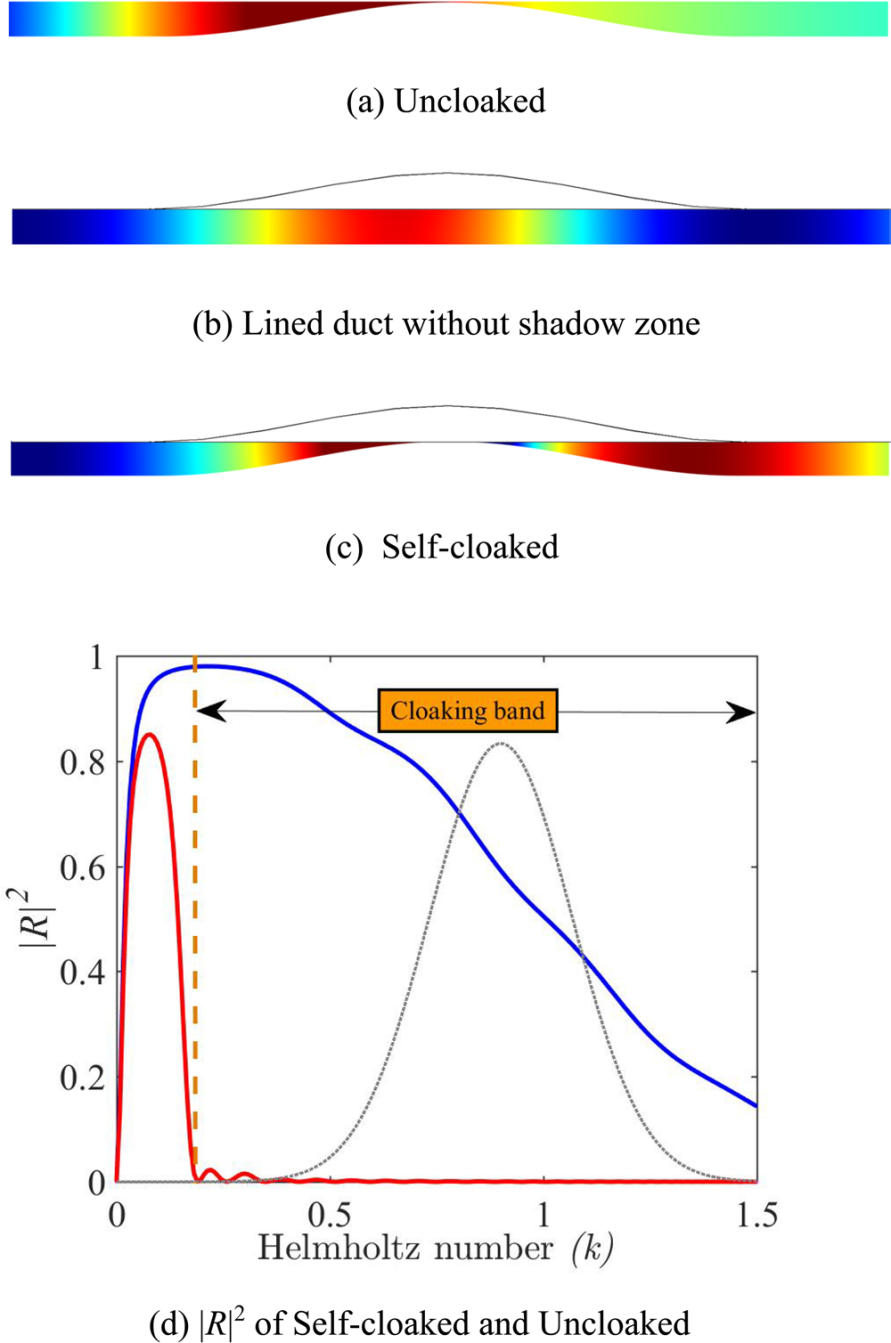
Visualization of self-cloaking phenomenon for a smooth cosine obstacle (*h*_0_ = 0.98, *b*_0_ = 1, *L* = 8.25), Real part of pressure fields at *k* = 0.25 for, (**a**) Uncloaked case, (**b**) Lined duct without shadow zone (**c**) Self-cloaked duct. (**d**) |*R*^2^| of a for uncloaked (blue) and self-cloaked (red) cases. The grey dotted line is the Fourier transform in arbitrary unit of the pulse used for time domain simulation.

**Figure 7:**
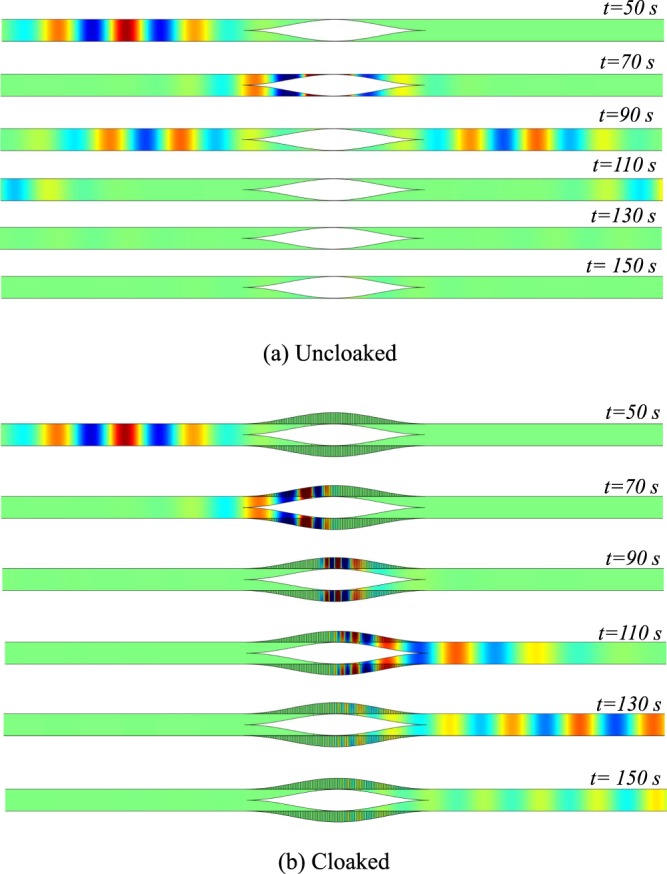
Time-domain results for Pressure field with Gaussian-shaped wave packet on the left side (**a**) Uncloaked, (**b**) Cloaked. The tubes as well as obstacle follow a cosine profile with *b*_0_ = 1, *L* = 8.25 and *h*_0_ = 0.98.

As a demonstration, an almost complete obstruction of the channel is considered even if the configuration is not very realistic due to the lack of losses consideration. The shape of the obstacle is then chosen to be:

5 $$h(x)=\frac{{h}_{0}}{2}(1+\,\cos (\frac{\pi x}{L}))$$

where *h*_0_ = 0.98 and *L* = 8.25. The height of the wall tubes is taken equal to the height of the duct. The results in the frequency domain for this geometry are given in Fig. 6. An almost complete reflection is obtained in the uncloaked case at low frequencies (*k* ≃ 0.25) as seen in Fig. 6(a). For such a low frequency, the admittance variation alone is unable to generate a significant shadow zone (Fig. 6(b)). When the smooth obstacle is added to the admittance (Fig. 6(c)), the reflection decreases drastically for the entire frequency band 0.25 < *k* < *π*/2 demonstrating the self-cloaking effect. The cloaking band is then considerably increased compared to sharp obstacles where this self-cloaking effect cannot be seen due to the reflection induced by any sudden change in the geometry. Snapshots of the pressure field are plotted in Fig. 7 when the duct is excited with a Gaussian wave packet (*k*_*c*_ = 0.9 & *s*_*t*_ = 6). Its Fourier transform is shown in Fig. 6(d). Compared to the case shown in Fig. 4, a significantly shorter wave-packet can be taken due to the extreme broadband nature of self-cloaking.

## Limitation of the Present Model

Two major limitations of the proposed model can be disturbing for the prediction of this in-duct cloaking effect. The first one is that we consider a continuous impedance instead of a set of discrete tubes with a width of *W*_*T*_ whereas *W*_*T*_ is not always small compared to the effective wavelength. The second one is that we have neglected the losses.

**Figure 8:**
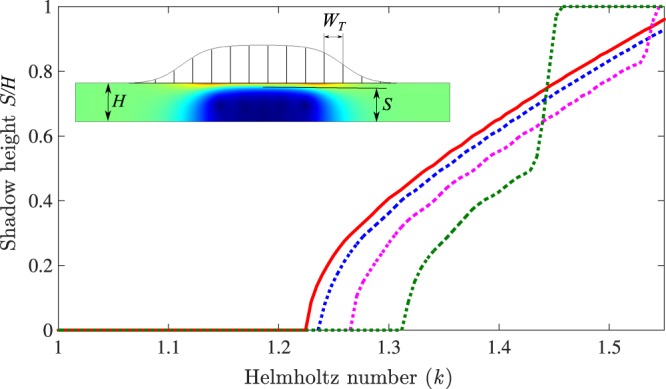
Variation of shadow height with the Helmholtz number *k*, computed with an admittance boundary *Y* (solid red) as well as using smoothly varying tubes (dotted) with tube width *W*_*T*_ = H/150 (blue), *W*_*T*_ = H/6 (magenta) and *W*_*T*_ = H/2 (green).

In order to study the effect of *W*_*T*_ on the cloaking effect, we have computed the height of the shadow zone for various *W*_*T*_. The shadow height *S* is defined as the maximal height for which the pressure is divided by 10 compared to the incident pressure. The normalized shadow height *S*/*H* is plot on Fig. 8 in solid red line when the impedance is considered and in dotted lines for several values of *W*_*T*_. On increasing *W*_*T*_, the assumption of smooth variation (*k b*_*T*_ ≫ 1, where *b*_*T*_ is the characteristic length of axial variation in tube height) is no longer valid for *k* ≈ 1.2, resulting in shift in the shadow height to higher frequencies. For large values of *W*_*T*_, there is a frequency at which the shadow height jumps to 1, which means that there is no further propagation in the cloaking zone and that the wave is fully reflected. Therefore, it is important to keep a small tube width to have the cloaking effect on the largest frequency band but in this case the loss effects can become significant.

**Figure 9:**
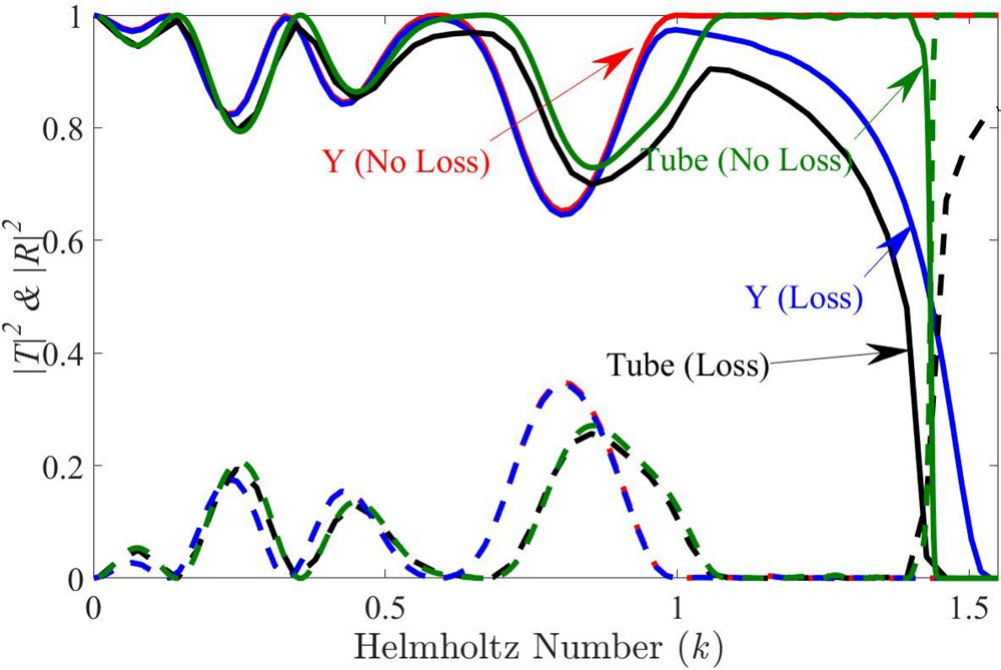
|*T*|^2^ (solid) and |*R*|^2^ (dashed) computed with admittance boundary *Y* (without/with) losses (red/blue) and with tubes (without/with) losses (green/black) for parameters *W*_*T*_ = *H*/2, *H* = 0.5 m, *f*_*r*_ = 170 Hz.

The viscous and thermal losses in a tube can be predicted using Kirchoff theory^17,18^. We consider in the following that the shear number giving the ration between the viscous shear layer and the tube width *W*_*T*_ is equal to 7 × 10^−4^ at the resonance frequency. The reflection coefficients *R* and transmission coefficients *T* are plotted in blue lines on Fig. 9 when an admittance with losses *Y* is used to cloak the triangular obstacle already considered in the previous sections. Compared to the lossless case (in red lines on the same figure), the reflection coefficient remains practically unchanged but, due to losses, the transmission is highly reduced near the resonance frequency of the tubes.

The combined effect of the finite width of the tubes and losses is also shown in black lines in Fig. 9 when *W*_*T*_ = *H*/2. These results are obtained by using the *Thermoviscous Acoustics* module of COMSOL. Compared to the case of tubes without losses (in green lines on the same figure), the reflection coefficient remains also practically unchanged with a jump to 1 in the cloaking band due to the large value of *W*_*T*_ used in the computations. The transmission |*T*|^2^ is reduced by the losses but remains around 0.8 on a significant cloaking range (*k* = 1.1 → 1.3) demonstrating that it is possible to realize this cloak in real life.

## Conclusion

In this paper, we construct a broadband acoustic cloak to reduce backscattering inside ducts using liner surface modes. By using a smooth variation in wall admittance of a duct which does not produce reflection, it is possible to create a silent zone in which acoustic waves do not penetrate. When an object is located in this silent zone, it has no influence on the wave propagation and this object is undetectable by acoustic waves. This means that the amplitude of the reflection coefficient of the object in the silent zone is equal to 0 and the amplitude of the transmission coefficient is equal to 1. This cloaking effect always exists for frequencies slightly lower than the liner resonance and the low frequency limit of this effect is related to the height of the obstacle. For an object that is half the height of the duct and using an admittance made of tubes of the same height as the duct, this effect occurs at frequencies between *f*_*r*_, the resonance frequency of the liner and 2*f*_*r*_/3, which is a very wide frequency band compared to other conventional cloaking techniques. When the object is smooth enough, the presence of the object helps the cloaking to occurs on a wider frequency bandwidth which is termed self-cloaking.

The novelty of this cloaking, made simply from a resonant wall, contrasts with other cloaking techniques that are more complex to perform and that only work at a single frequency. The creation of a zone of silence in a duct and the concentration of the wave along a wall can also have applications other than cloaking, such as the realization of an anechoic termination^19^. The cloaking works with inclusion of losses as well, but with reduced frequency range.

## Supplementary information

Demonstration of cloaking a scatterer

Demonstration of Self-cloaking
