# Supplementary figures and images for: Using liner surface modes in acoustic ducts to make obstacles reflectionless
Source: Sci Rep. 2019 May 6;9:6981. doi: 10.1038/s41598-019-43538-3 (PMC6502847; doi:10.1038/s41598-019-43538-3)

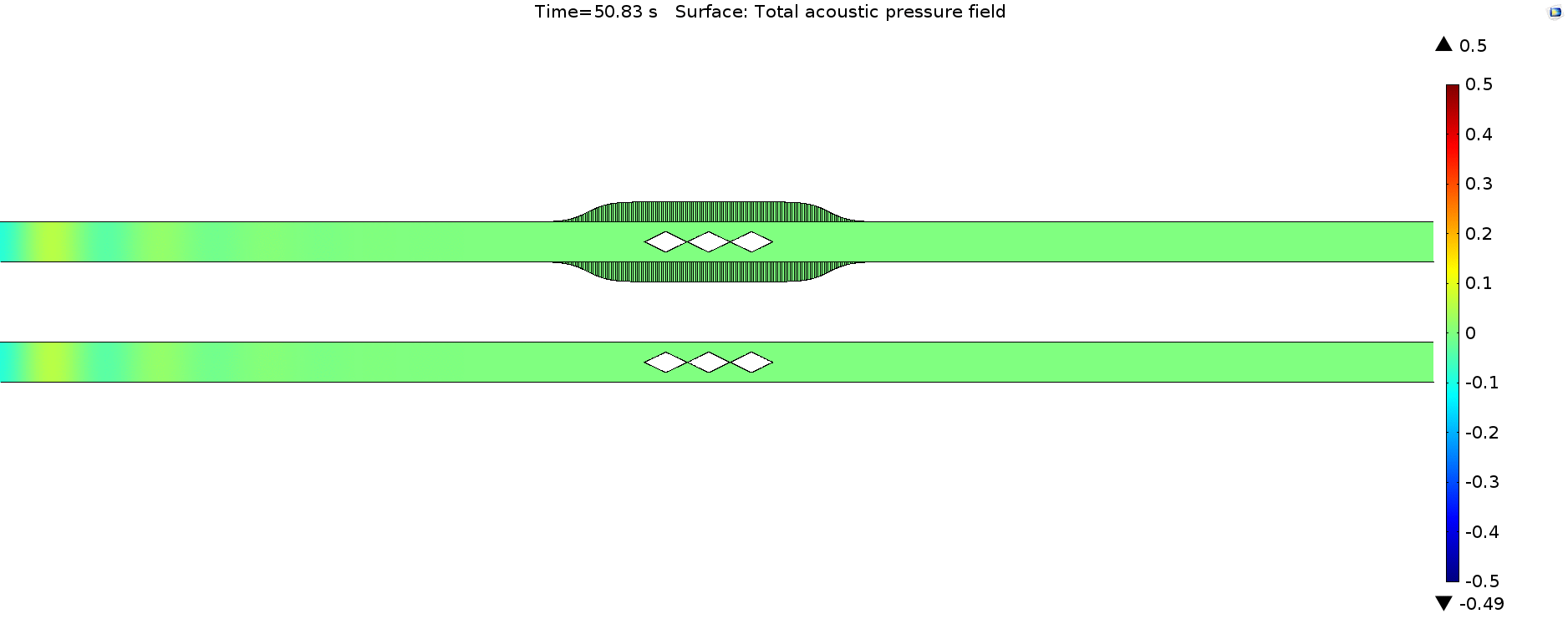

Supplement: Supplementary file 1 — Demonstration of cloaking a scatterer [file 41598_2019_43538_MOESM1_ESM.gif]

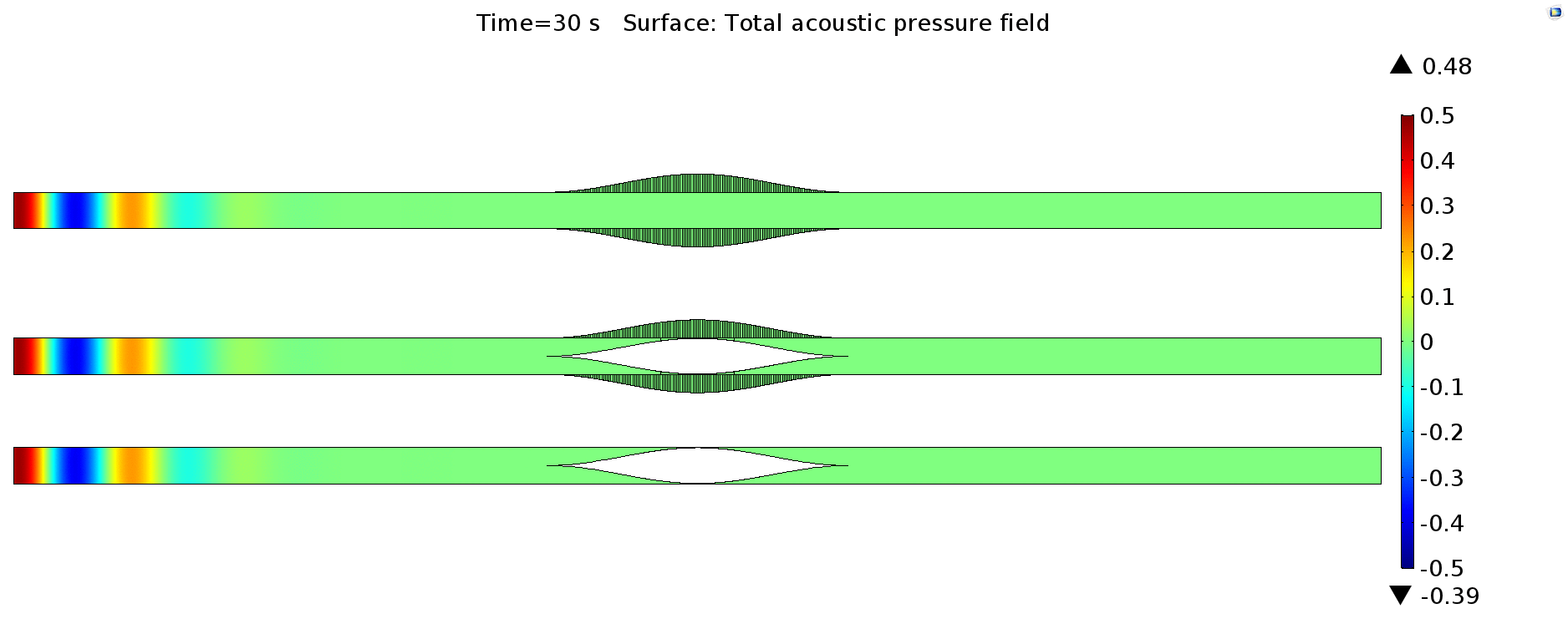

Supplement: Supplementary file 2 — Demonstration of Self-cloaking [file 41598_2019_43538_MOESM2_ESM.gif]
